# Mental Health and Addiction Services Exclusive to LGBTQ2S+ during COVID-19: An Environmental Scan

**DOI:** 10.3390/ijerph19105919

**Published:** 2022-05-13

**Authors:** Michael Chaiton, Rebecca Billington, Ilana Copeland, Luc Grey, Alex Abramovich

**Affiliations:** 1 Dalla Lana School of Public Health, University of Toronto, Toronto, ON M5T 3S7, Canada; alex.abramovich@camh.ca; 2 Centre for Addiction and Mental Health, Toronto, ON M5S 2S1, Canada; rebecca.billington@mail.utoronto.ca (R.B.); lani.copeland@mail.utoronto.ca (I.C.); luc.grey57@gmail.com (L.G.); 3 Department of Psychiatry, University of Toronto, Toronto, ON M5T 3S7, Canada

**Keywords:** mental health services, access to care, LGBTQ, COVID-19

## Abstract

Background: Youth who are lesbian, gay, bisexual, trans, queer, 2-spirit, and of other identities (LGBTQ2S+) experience mental health disparities and higher rates of substance use when compared to their cisgender and heterosexual peers and yet also experience more barriers to access to services. The purpose of this paper is to determine the types of mental health and substance use programs and services exclusive to LGBTQ2S+ youth in Ontario during the pandemic. Methods: An environmental scan was conducted to identify existing programs and services in Ontario, Canada that offered exclusive mental health and addiction services to LGBTQ2S+ individuals aged 16–29, either by offering services to all or subgroups within the population. Organizations, services and programs were classified by the geographical distribution of services, populations served, types of programming or services, methods of service delivery, and program criteria. Results: In total, 113 organizations and 240 programs and services were identified as providing mental health and substance use services exclusively to LGBTQ2S+ youth. Identified adaptations for the COVID-19 pandemic included cancelling in-person services, increasing online and telephone services, and expansion to province wide from local availability. Conclusions: The findings highlight the importance of offering services that provide culturally inclusive care for LGBTQ2S+ youth, and these results can also be used by policy makers to inform policies. In particular, there was a lack of culturally relevant clinical services for youth requiring a greater intensity of treatment.

## 1. Background

As a result of the COVID-19 pandemic and preventive measures such as social distancing, an “echo pandemic” of declining mental health amongst the Canadian population has emerged [1] The COVID-19 pandemic is exacerbating mental health challenges and widening service gaps, especially among those in marginalized groups and those with pre-existing mental health issues [2,3]. Research on mental health during the pandemic has found that 40% of Canadians stated their mental health has declined due to COVID-19 and 48% of individuals felt anxious or worried [4,5]. Even prior to the pandemic, the burden of mental illness was widespread in the population. Worldwide, an estimated 264 million people are affected by depression alone [6]. Mood and anxiety disorders are the most prevalent mental health issues in Canada, occurring among 11.6% of Canadian adults [7]. Our mental health care system is overwhelmed and too underfunded to address the pervasiveness of mental health challenges [8]. At the same time, many people are reluctant to maintain or improve their mental health, with a stigma-based perception that mental health is only an issue for people with a diagnosed mental disorder, rather than a concern for everyone [9].

In Canada, provincial governments are responsible for mental health treatment, although financing for individual programs, notably non-clinical care, might come from federal, provincial, or local sources [7]. Professional mental health and addiction services include medication and counselling. Family doctors, psychiatrists, psychologists, nurses, and other mental health practitioners can give care in hospitals or in the community as well as peer-led support groups.

The capacity to obtain medical treatment and services when required, in a secure, culturally competent, and appropriate setting for the individual, is referred to as service accessibility. There is a well-documented demand for mental health and addiction care; and in 2018, an estimated 5.3 million Canadians sought care for their mental health [10,11]. A total of 1.2 million (22 percent) of these 5.3 million people indicated that their requirements were only partially satisfied, while 1.1 million (21 percent) claimed that their needs were completely unmet. The need for medicine was the most likely to be satisfied (85%), while the need for counselling was the most likely to be unmet (34%). Furthermore, excessive wait periods limit access to services [12].

Access to mental health and addiction treatments in Canada has been hampered by a large number of barriers [10]. These include long wait times, a scarcity of accessible mental health professionals, a lack of mental health service integration and government supervision, cultural and linguistic barriers, inequities due to geography or demographics [e.g., youth, rural communities, and Indigenous populations], and the cost of services [13,14,15,16,17].

Personal circumstances (78.2%), such as not knowing where to get help, being too busy, or not being able to pay for treatments, were the most prevalent barriers stated by the 2.3 million Canadians who reported unmet or partially completed mental health care criteria [10]. Stigma, however, is a primary barrier for Canadians with mental health disorders, who report feeling uncomfortable talking about their problems in general or feeling disrespected by health professionals [18].

In particular, stigma affects youth (defined as 16–29 in this paper) who are lesbian, gay, bisexual, trans, queer, 2-spirit, and of other identities (LGBTQ2S+) who experience greater mental health disparities and higher rates of substance use when compared to their cisgender and heterosexual peers and consequently have higher needs for services [19,20,21,22,23]. A systematic review by Plöderl and Tremblay [19] suggests that 97% of reviewed adolescent studies indicate higher rates of depression among LGBTQ2S+ youth, and 98% of studies indicate higher rates of adolescent suicidality in this population. In addition, all studies that explored anxiety indicated disproportionate rates of mental health issues for LGBTQ2S+ youth. The research also indicates higher rates of post-traumatic stress disorder [20] and non-suicidal self-injury [21] among LGBTQ2S+ youth. A meta-analysis by Marshal et al. [23] illustrates that youth sexual orientation was associated with higher rates of lifetime alcohol, cannabis, and tobacco use, as well as lifetime illicit and injection drug use. Similar findings emerged through a cross-sectional study exploring transgender youth substance use, where transgender youth had higher rates of substance use, past 30 day use, and earlier age of onset for cannabis use compared to their cisgender peers [22]. Research has also shown that ethnicity and race influence mental health outcomes, where Black and Hispanic sexually diverse youth were significantly more likely to report suicidality compared to their White, heterosexual peers [24].

Several structural mechanisms have been identified as risk and protective factors among LGBTQ2S+ youth. Minority stressors, such as harassment, discrimination, violence, and internalized stigma, were found to be positively associated with negative mental health outcomes for lesbian, gay male and bisexual youth as identified in a meta-analysis by Dürrbaum and Sattler [25]. Mereish and Miranda [26] supported this finding by demonstrating a causal relationship between cravings for alcohol and sexual stigma exposure through an experimental design with LGBTQ2S+ youth. Lack of family support is also associated with higher emotional distress for LGBTQ2S+ youth, even when controlling for victimization and overall social support [27]. Several factors such as feeling connected to parents and other adults in one’s community as well as feeling safe at school have been identified as protective against depression and suicidality for LGBTQ2S+ youth [28]. Community supports have also been identified as a protective factor, where the presence of events, community resources, and organizations that offer programming specific for LGBTQ2S+ community members is associated with lower rates of substance use, suicide attempts, and self-harm behaviours among LGBTQ2S+ youth [29,30,31].

Despite experiencing higher rates of mental health and substance use, LGBTQ2S+ youth also report a greater likelihood of not having their needs met through treatment. Accessible treatment requires both the existence of a program and that program having the ability to provide safe and affirming care. Burgess et al. [32] identified that people in their LGBTQ2S+ sample were more likely to report having unmet mental health needs compared to the heterosexual comparison group within the past year. This is consistent among transgender individuals, where an Ontario-based cross-sectional study by Steele et al. [33] found that transgender women were 2.4-fold more likely to report having unmet mental health needs compared to cisgender women. In a study of addiction treatment outcomes and experiences using a convenience sample of 81 men from the LGBTQ2S+ community, compared with 55 heterosexual men, men from the LGBTQ2S+ community reported lower levels of connection during treatment, and were more likely to identify “needs not met” as the main reason for ending treatment early [34].

There are several barriers to accessible care for LGBTQ2S+ youth that have been identified in the literature that have led to unmet needs. A large-scale study of mental health service utilization (use of services) across three post-secondary institutions in California found that students who identified as LGBTQ2S+ were significantly more likely to report barriers towards accessing on-campus mental health support, and significantly more likely to report multiple barriers [35]. It is also reflective of an analysis conducted by Lyons et al. [36] of the results for three open prospective cohort studies of individuals who used illicit drugs in Vancouver. This analysis identified that a higher percentage of LGBTQ2S+ women reported barriers accessing treatment (12.7%) compared to the heterosexual sample (7.7%). Several barriers to mental health care were identified in a qualitative study of 104 youth, where system-level barriers (i.e., lack of competence to provide affirmative care, dominance of medication, service availability and accessibility, and previous experiences), sociocultural barriers (i.e., stigma, not wanting parents to know, and lack of family support), and individual barriers (i.e., beliefs about the severity of need, beliefs about ability to cope, and lacking self-confidence) were identified [37].

For some members of the LGBTQ2S+ community, these barriers appear to be exacerbated by the conditions of COVID-19, where individuals were significantly less likely to access telehealth for mental health support during the pandemic, while being more likely to be connected to a mental health professional [38,39]. This research indicates that there is a greater need for mental health services targeted to LGBTQ2S+ youth; however, the research also shows that there are also greater barriers to accessing care. Different social locations intersecting with gender diversity and sexuality also influence experiences within the mental health system. For example, a qualitative study of Black and Hispanic LGBTQ2S+ young adults identified several additional barriers, including stigma associated with mental health symptoms and sexuality, ambivalence about the efficacy of treatment, and lack of family support due to family’s previous negative experiences with mental health interventions [40]. We focus in this paper on dedicated services for LGBTQ2S+ which have been implemented specifically to address the needs of the LGBTQ2S+ populations and provide culturally appropriate and affirming care. While the effectiveness of these programs in providing quality care may vary, they provide accessibility to LGBTQ2S+ individuals to services where they can expect safe and affirming care.

The literature has acknowledged the mental health disparities, unmet mental health needs, unique barriers to care and added potential vulnerabilities for LGBTQ2S+ youth in the context of the COVID-19 pandemic. Specifically, the literature has identified that existing structural disadvantages and vulnerabilities have been exacerbated in the context of COVID-19, where 47% of LGBTQ2S+ individuals indicated that COVID-19 has had a significant impact on their mental health, compared to 26% of the national average [41]. It has also been identified that mental health and substance use services exclusive to LGBTQ2S+ youth are well positioned to address these disparities in a culturally informed and safe way. However, little is known about the current state of these programs and services within Ontario’s mental health and addiction system, nor what adaptations have been made during the pandemic in response to public health regulations and recommendations. Therefore, the purpose of this environmental scan is to determine the types of mental health and substance use programs and services exclusive to LGBTQ2S+ youth in Ontario in the context of the COVID-19 pandemic.

## 2. Methods

### 2.1. Environmental Scan

The purpose of pursuing an environmental scan was to determine which services currently exist specifically for LGBTQ2S+ youth in Ontario, as well as to understand how these services may have been adapted within the context of the COVID-19 pandemic. An environmental scan is a methodology developed for business strategic purposes that has been adapted for other contexts including assessment of availability and access to health care [42]. For the purposes of this paper, the term LGBTQ2S+ youth refers to individuals between the ages of 16 and 29 years of age who identify as members of the LGBTQ2S+ community. It was necessary to first determine the current state of services for LGBTQ2S+ youth in Ontario, in order to move forward with trying to understand how to decrease barriers and increase access to mental health and substance use services for LGBTQ2S+ youth.

### 2.2. Search Process

This environmental scan intended to identify existing programs and services in Ontario that offered exclusive mental health and addiction services to LGBTQ2S+ individuals, either by offering services to all or subgroups within the population. A set of inclusion criteria were established to determine eligibility of the programs and services to be included in the scan. The programs and services were required to serve youth (ages 16 to 29), either specifically or as part of the broader adult population. In addition, the services were required to offer support or interventions for mental health or substance use, either as an entry point for exposure to the mental health and substance use treatment system or by directly offering treatment. Private mental health practitioners and psychotherapists were excluded from this scan; however, organizations and networks of several private mental health practitioners and psychotherapists that offered services specifically for LGBTQ2S+ clients were included.

The list of programs and services were compiled through an Internet search leading to several Ontario-based service databases (*n* = 5), resource guides (*n* = 4), and a Facebook group (*n* = 1). Initially, each resource was scanned for mental health and substance use programs and services exclusive to LGBTQ2S+ individuals. This information was captured in an Excel spreadsheet. Each program and service’s website and/or social media pages (Facebook, Twitter, or Instagram) were manually reviewed for inclusion criteria. All resources were identified for inclusion between November 2020 and February 2021.

### 2.3. Analysis

Once programs were identified as meeting the inclusion criteria, the environmental scan extracted the following information from the identified programs and services: organization that offers the program/service; name of program; location; setting where services are offered (e.g., community, hospital, and residential treatment); whether the organization and/or program offered exclusive services to LGBTQ2S+ populations; target population for the program/service (e.g., post-secondary students and Indigenous Peoples); target age range for the program/service; program topic focus (e.g., mental health or addiction/substance use); relationship to mental health/addiction system (e.g., entry point into the system and therapeutic intervention); type of program (e.g., peer support group and counselling); cost; method of program delivery during the COVID-19 pandemic and website.

## 3. Results

**Figure 1:**
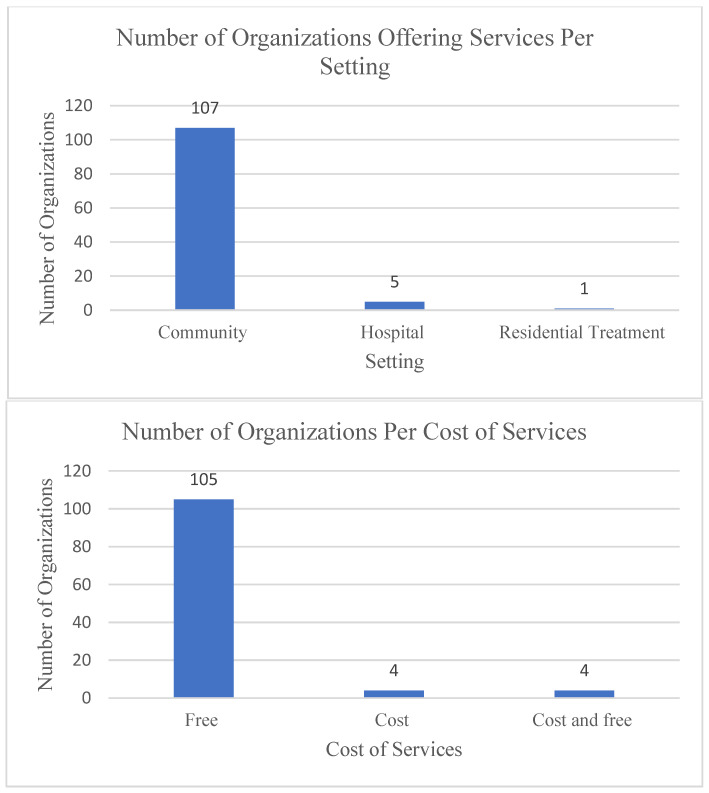
Number of organizations offering services per setting and per cost of services (*n* = 113).

In total, 113 organizations and 240 programs and services were identified as providing mental health and substance use services exclusively to LGBTQ2S+ youth (Figure 1). Four main themes were identified from the scan, including the distribution of services, types of services, methods of service delivery and program criteria. In addition, there were various adaptations identified in light of the COVID-19 pandemic, which are described below as they relate to each of the four themes.

### 3.1. The Geographical Distribution of Services

One goal of this environmental scan was to identify the distribution of services across Ontario as a means of exploring accessibility. The scan identified that most services were offered in a community setting. Only one full mental health program was offered through a hospital or residential treatment setting, where others that took place in these settings offered a particular group or service within a broader program. The distribution of programs and services per location was also identified. As identified in Table 1, regions of Local Health Integration Networks (LHIN) were used to categorize locations. LHIN regions were selected due to their role in the distribution of health care spending and provisions across the province, their ability to capture distinct population centres, and the availability of region-specific Canadian census information for further analysis. When looking at the distribution of services relative to population density, the North West LHIN region was identified as having the most services per 100,000 people at a rate of 5.26, followed by Toronto Central at 3.57, with Central having the least service per population at 0.94. When looking at the distribution of services relative to geographical distribution, the Toronto Central LHIN region had the most services per 100 square kilometers at a rate of 22.90, over 13-fold the next highest number of services, with Mississauga Halton at 1.71, and much higher than the lowest rate per LHIN, with North East at a rate of 0.0028. For those that are seeking mental health or substance use treatment options that are specific for LGBTQ2S+ individuals, options are few and they decrease in frequency the further they are from the Greater Toronto Area. Removing Toronto-based services from the tabulations resulted in almost a 3% decrease in mental health and substance use interventions for this population.

### 3.2. Types of Services

**Figure 2:**
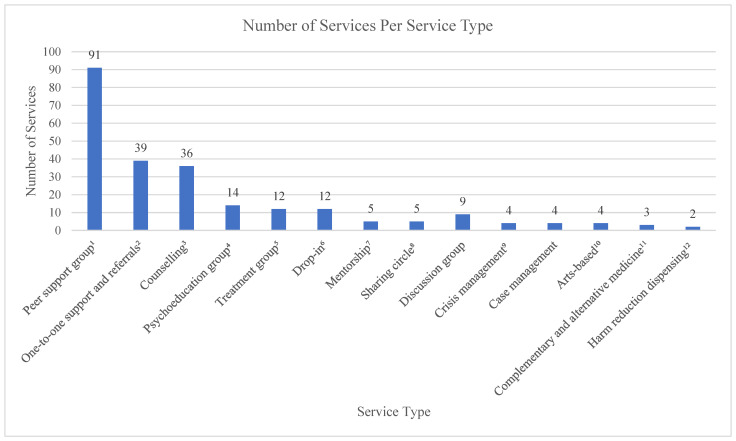
Number of services per service type, service focus, and target population (*n* = 240). ^1^ Category includes social and support groups; support groups; peer wellness groups; drop-in support groups; peer support and social groups; social support groups; peer support groups; social groups; social and peer supports; social support groups with referrals and strategies; social support drop-ins; social groups with health and wellbeing promotion; social support groups with topics of mental health and harm reduction; peer support and referral groups; peer support drop-in groups; groups and events that offer support; drop-in social groups; social support groups with referrals; self and collective care groups. ^2^ Category includes weekly check-ins; one to one peer support; peer support and referrals; social service and health referrals; peer support navigator; support and referrals; mental health navigation; referrals; community resource navigation; drop-in with one-to-one mental health support available; one-to-one support; mental health navigation; facilitate access to services and help self-define wellness; system navigation and supportive listening; advocacy, support, and referrals; one-to-one support and referrals; peer-to-peer monthly support and counseling; individual support; support and referral online chat; active listening sessions; resource referral; active listening; community resources; staff support; referral and support line; peer support helpline; peer support; support line; peer support warm-line (for non-crisis support). ^3^ Category includes counselling; collectives of several private practice counsellors; mental health therapist; short-term, individual, couple and family counselling; individual, couple, family counselling; counselling and referrals; six session counselling; single-session, solution-focused counselling; limited ongoing counselling; walk-in counselling. ^4^ Category includes psychoeducation and social support groups; psychoeducation and peer support groups; workshops; workshop/psychoeducation groups; support and education groups; wellness groups; body image groups; meth abstinence and sex groups; workshops with topics on mental health; educational workshops; mental health and wellbeing workshops. ^5^ Category includes 12-step groups; interpersonal trauma groups; structured relapse prevention groups; interpersonal groups; emotional skills groups; mindfulness groups; coping skills groups; mental health groups; emotion-focused mindfulness groups; harm reduction groups; trauma groups; CBT-based groups; trauma counselling groups; DBT skills groups. ^6^ Category includes drop-ins; drop-ins with one-to-one mental health support available; drop-in centres; drop-in and social spaces with resources. ^7^ Category includes mentorship; peer mentors; peer mentoring. ^8^ Category includes talking circles; family, friend or relationship circles; sharing circles; care circles. ^9^ Category includes crisis management; crisis intervention and emotional support; crisis and peer support hotline. ^10^ Category includes zines; de-stress art groups; arts-based workshops; arts collaborations. ^11^ Category includes ecotherapy; trauma sensitive yoga; retreat-style workshop. ^12^ Category includes harm reduction dispensing; harm reduction education and dispensing.

Most population-based mental health and substance use services geared towards LGBTQ2S+ youth in Ontario were either free or included some free aspects (Figure 2). This is to be expected as individual private mental health practitioners were excluded from the environmental scan. Peer support groups were the most frequently available form of support at 91 of all specific services. Peer support groups along with several other modalities of services operate as entry points into the mental health system, consisting of 78.33% of services across the province. These are valuable services where individuals can be introduced to many topics around mental health and receive education around treatment options and referrals. They are also important sources of support for the social and cultural domains of mental health through sharing of common experiences and the recognition of social and political determinants of health, which can lead to the alleviation of shame and improve self-acceptance. For those that experience added layers of disenfranchisement, they can also be a valuable source of meeting basic needs such as food for those with limited financial resources, or information sharing around navigating the health care or immigration systems for those that are newcomers to Canada [43].

There is also a dearth of programming related to substance use across the province, as only one program was identified. Most services that did offer substance use support did so in conjunction with mental health interventions, which may pose as a barrier to care for those without concurrent mental health concerns or who are experiencing stigma-related perceptions of mental health.

### 3.3. Methods of Service Delivery

**Figure 3:**
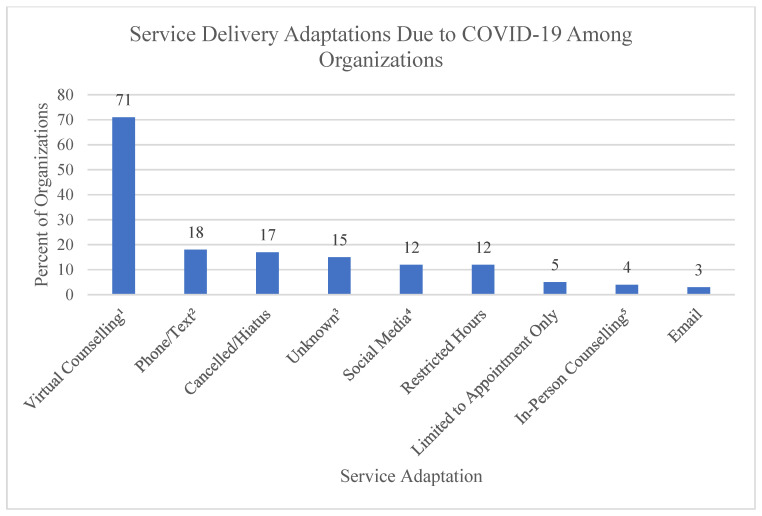
Service delivery adaptations among organizations (*n* = 113). ^1^ Virtual counselling includes any form of counselling that is identified as taking place in a virtual or online setting, such as Zoom. ^2^ Phone/Text refers to the use of either phone calls or text messaging as a means of service delivery. ^3^ Unknown refers to programs that did not specify the adaptations made to their services. ^4^ Social media refers to the use of social media platforms such as Facebook, Instagram or Discord as an adaptation to service. ^5^ In-person counselling refers to programs where no adaptations or changes were identified to the in-person counselling services.

Most services have moved online with a variety of different formats. This means that out of the 113 services, many of them were categorized into multiple adaptation categories, as shown in Figure 3. Virtual counselling, such as through Zoom, was the most common platform used. However, others such as Discord, Skype, Cisco Webex, Olark, tawk, and Google Meet were also identified. Social media sites such as Facebook and Instagram were identified as ways of maintaining engagement through sharing resources, interactive activities, hosting live events, or used to chat with community members seeking support. Several organizations implemented a “check-in” program, where staff would reach out once a week via email, text, or social media to remain in contact with those facing increased isolation during this time. Other creative ways that organizations attempted to meet community needs during the pandemic include offering socially distanced walks, offering monthly kits for online group participation, and offering new online support or treatment groups in response to the needs of the community during COVID-19. Several programs increased their service areas due to their shift to online formats and no longer requiring travel to a centralized area to receive services. Some programs expanded to province or nationwide, and others were better able to serve those living on the periphery of their service area.

The pandemic has also created many limitations to the mental health and substance use system. Seventeen percent of organizations reported cancelling all or parts of their services that were not adapted for online formats. Walk-in, drop-in, and in-person programs adapted by moving to either online-only formats, appointments only, reducing the number of people allowed in a space, requiring masks for in-person services, limiting the amount of people accessing in-person services to those urgently in need, and/or providing time limits for in-person services. Some counselling programs stopped accepting new participants related to the increased needs of current clientele and resource constraints. It is also important to note that it was not possible to identify current service delivery methods on websites or social media for 17 services. Not updating websites with service disruptions may operate as a barrier to care related to accessibility needs as described in the *Accessibility for Ontarians with Disabilities Act* [44].

### 3.4. Program Criteria

**Figure 4:**
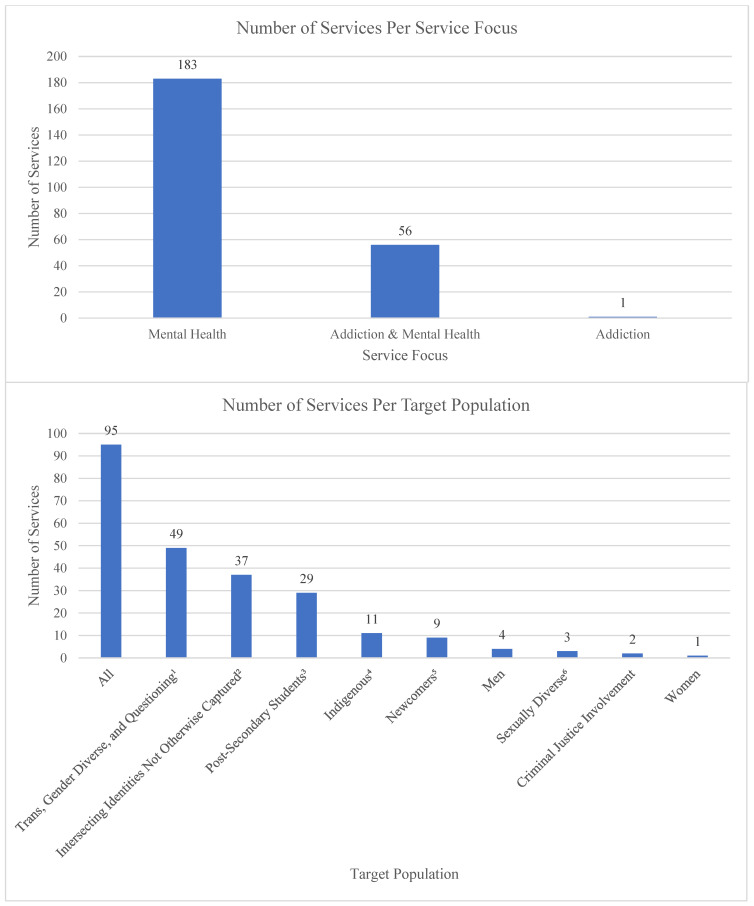
Number of Services per Service Focus and Target Population (*n* = 240). ^1^ Category includes trans, non-binary, and/or gender non-conforming; gender expansive, trans and non-binary identities; transgender and gender diverse; trans and gender diverse; trans and non-binary-identified; trans and gender non-conforming; trans, two spirit, gender non-conforming, or otherwise not cisgender; trans, two-spirit, and gender-diverse; trans and gender-independent; two-spirit, trans, gender non-binary, gender-queer, and gender non-conforming; trans, non-binary, and two-spirit; trans, non-binary and gender-diverse; trans, non binary and two spirit people; trans/2-spirit and non binary; transgendered and gender non-conforming; gender diverse; experiencing gender dysphoria; gender diverse, trans, non-binary and questioning; experiencing or questioning changes on the gender spectrum; trans, non-binary, gender non-conforming and gender questioning; transgender and those struggling with gender identity issues; trans men, trans women, non-binary, gender variant folk, and people who are questioning; trans, nonbinary, genderfluid, or who are questioning their gender identity; transgender; trans; transgender and questioning; trans and questioning; Transgender women; Non-Binary; Transmasc. ^2^ Category includes people labelled with intellectual disabilities; Women who are black, indigenous, queer, trans, people of colour; People who identify as both Muslim and queer/trans; East and Southeast Asian identity; Indigenous ace community members; Francophone; Living with a mood disorder; south Asian guys who like guys; Bi+ men (including trans men) and non-binary folx; Bi+ women (including trans women) and non-binary folx; East and Southeast Asian men; Black African Caribbean Trans/2Spirit and Non Binary; Youth who use substances; Gay or bisexual men living with Human Immunodeficiency Virus (HIV) Black Trans women and Non-Binary people; Tamil men who like men; People from diverse cultures; Gender and sexually diverse refugees living in Turkey; black gay, bisexual, queer, questioning, trans* and other men who have sex with men (MSM); black guy into guys; QTBIPOC (Queer and Trans Black, Indigenous, and Person of Colour); Queer and Trans BIPOC survivors of sexual and gender based violence. ^3^ Category includes post-secondary students; post-secondary trans and non-binary students; post-secondary transgender and/or non-binary students; post-secondary students who are trans, two-spirit, non-binary or other gender diverse people; Post-secondary students who are women who are also attracted to women; Post-secondary and bisexual, pansexual, polysexual, and other mspec students; Post-secondary students who identify as disabled or a person with a disability; Post-secondary and international students; Post-secondary and trans students; Post-secondary black 2STLGBQIA+ students; Post-secondary students identifying with or experiencing social anxiety, shyness, and awkwardness; Post-secondary students who are 2STLGBQIA+ Black, Indigenous, and People of Colour; Post-secondary students who are men who are also attracted to men; Post-secondary and asexual and/or aromantic students. ^4^ Category includes 2-spirit, including First Nations, Metis and Inuit; two-spirit and Indigenous; two-spirit and trans Indigenous; Indigenous; two spirited; 2S trans Indigenous; two spirit and trans Indigenous. ^5^ Category includes newcomers, African, Caribbean and Black (ACB) newcomers. ^6^ Category includes sexually diverse; bisexual, pansexual, bi-curious, and questioning; bisexual, pansexual, 2 spirit, fluid and other non-monosexual people, and those questioning.

There is much diversity among LGBTQ2S+ youth, where the intersection of identities and social locations influences experiences in unique and important ways. All programs included in this environmental scan offered exclusive programming to LGBTQ2S+ individuals; however, many programs also considered additional social locations within LGBTQ2S+ experiences, offering programming to meet identified needs within the community (Figure 4). It is important to note within programming for transgender and gender-diverse individuals that there was a notable inconsistency with language used to describe the target population of programs, where some programs made no mention of non-binary and gender-diverse identities, or those who were questioning their identities. As it is beyond the scope of this environmental scan to reach out to the programs individually to confirm whether this was an oversight or intentional specific programming, programs were categorized based on the information listed on their websites or social media. Other specific social locations considered within programming included cultural, religious, or ethnicity related criteria (44 services); post-secondary enrollment (29 services); sexuality related criteria (22 services); physical, cognitive, developmental, and mental health-related abilities (7 services); and criminal justice involvement (2 service). Many programs also identified age criteria for participants. One note is that although only 1 service was identified for young adult-specific programming, 29 services were identified specifically for post-secondary students. Although no age criteria were listed for post-secondary students, many are young adults and therefore these spaces may offer programming targeted to this demographic. A final note regarding target populations of services is that several offered support to multiple, intersecting social locations, further recognizing the diversity of experiences.

## 4. Limitations

There are several limitations in this study, including that the information extracted from websites and social media was assumed to be accurate and up to date. We did not have data from prior to the COVID-19, which limited the inferences possible on service adaptation and the ability to compare rates pre- and post-epidemic. Certain programs also had inclusion criteria that were ambiguous and may have benefitted from further clarification regarding the target population for their service. In addition, private mental health services and services that offered welcoming or affirming programming without exclusively serving LGBTQ2S+ youth were excluded from the scan, meaning that the results of this scan may not include the entirety of mental health and substance use services available to LGBTQ2S+ youth in Ontario.

## 5. Discussion

This scan was able to discern the mental health and substance use services available for LGBTQ2S+ youth in Ontario, as well as certain adaptations that were put in place due to the COVID-19 pandemic. Although there are currently some specific services available for LGBTQ2S+ youth, more work needs to be conducted to ensure accessibility to these services in Ontario. For example, this scan identified that many of the available services were in the Toronto Central Region and that most services were being offered within community settings. Services that specialize in LGBTQ2S+ populations are needed to ensure accessibility of culturally safe and affirming care to minimize the barriers associated with homophobia and stigma, particularly for youth from diverse cultural backgrounds.

However, as identified in this research, there are also many barriers that have arisen during the pandemic, making mental health and substance use services less accessible for LGBTQ2S+ youth. It will be important for future policies and practices to address these barriers, to subsequently increase access to affirming care in this population. Finally, most of the available services were offered through peer support, which is an important consideration regarding the hiring practices of mental health and substance use organizations. It will also be important for organizations to consider the additional need for clinical counsellors to support individuals from this community, as the scan highlighted a lack of necessary counselling support to meet the needs of LGBTQ2S+ youth. The themes identified from the scan should be used to inform future policy and practices in terms of providing culturally appropriate care to LGBTQ2S+ youth.

These results are also consistent with the literature on this topic, which suggest that certain individuals may be differentially affected by the psychological impacts of the COVID-19 pandemic [38]. For example, the results from the 2SLGBTQ+ Commercial Tobacco Project Screening Questionnaire, a survey administered to youth in Ontario and Quebec who identify as gender or sexually diverse, showed that 75% of respondents sought help for their mental health and faced barriers to accessing services [45]. Individuals who faced barriers such as discrimination or decreased social support before the pandemic, including LGBTQ2S+ youth, may experience exacerbated psychological effects as a result of the COVID-19 pandemic [38]. Further, this scan amplifies a body of evidence that indicates the importance of increasing the availability of mental health and substance use services for LGBTQ2S+ youth and decreasing barriers that exist for this population to access services.

Kurdyak and colleagues point out that, while certain areas of Ontario have a deficit of psychiatrists and psychotherapist physicians, others, such as the cities of Toronto and Ottawa, have a surplus [46]. Furthermore, not all psychotherapy is equally helpful—some modern psychotherapy is of poor quality, unstructured, and may not be evidence based [47]. Peer groups, the most accessible option for LGBTQ2S+ youth seeking care, are even more unlikely to be evidence based. More services providing evidence-based care specific to LGBTQ2S+ youth are urgently required.

This scan also distinguished the difference in access for individuals based on program criteria. The results of the scan showed that although certain services were geared towards LGBTQ2S+ youth, they had additional inclusion criteria that may be limiting for individuals. In other words, the services identified in the scan are not accessible by all LGBTQ2S+ youth due to additional criteria reflective of intersecting identities. Although this allows for the recognition of intersecting social locations within the community, it also speaks to a dearth of exclusive services as the number of services is smaller than it initially appears. When considering the methods of service delivery, it was discovered that certain programs are now able to offer services province wide due to the increase in remote services being offered during the pandemic. Prior to the pandemic, there were limited services offering programs to particular ethno-racial groups across the entirety of Ontario. Therefore, funding to support the expansion of these programs to virtual services might offer an opportunity to connect individuals with similar social locations outside of a centralized area for their mental health and substance use care. Ultimately, this may provide the opportunity to increase access to culturally sensitive services for LGBTQ2S+ youth who would not otherwise have access to these services within their geographical region.

The literature offers several strategies that the mental health system can implement to address the identified disparities and offer culturally informed care. One way to do this is by offering affirming care. This involves clinician inquiry around gender and sexuality and addressing the mechanisms by which mental health disparities are fostered, such as by normalizing the impacts of minority stress and restructuring related cognitions [48,49]. There are limitations to this approach, however. Individual clinician competency can vary, and as youth may engage with multiple service providers within a given organization, there is risk or perception of risk of having their identity stigmatized by providers, which may result in youth not disclosing their identity or acting as a barrier to seeking out support [40]. There can also be risk or anticipation of risk of experiencing discrimination and harassment from other participants such as in the waiting room or in group settings, which may act as a barrier to care [50].

For those who are unable to meet their needs through general mental health practitioners offering affirming care, the literature has identified mental health and substance use services exclusive to LGBTQ2S+ youth as a means to better address disparities. A pilot study conducted by Craig and Austin [51] looked at the impact of LGBTQ2S+ youth-specific group cognitive behavioural therapy (Project AFFIRM) on experiences of depression, noting a significant decrease in depression at post-test sustained at 3 month follow up. A randomized control trial of group cognitive behavioural therapy for sexually diverse young adult men found similar results, where, compared to waitlist control, participants reported a significant decrease in depressive and anxiety symptoms as well as alcohol use problems and past 90 day heavy drinking [52]. Encouraging results of targeted care were also described by Senreich [34], where when sexually diverse men received substance use treatment exclusive to LGBTQ2S+ participants, a significant increase in program completion, abstinence following treatment, and interpersonal connection was noted to the point that all significant outcome differences between sexually diverse men and heterosexual men disappeared. Interventions exclusive to LGBTQ2S+ youth hold an important place in the mental health and substance use system to respond to the specific needs of this group.

## 6. Conclusions

This environmental scan sought to identify the landscape of mental health and substance use programs and services exclusive for LGBTQ2S+ youth in Ontario during the COVID-19 pandemic. Several themes were identified, including the distribution of services, types of services, methods of service delivery, and target populations served. These results have important implications for mental health and substance use policy and practice considerations. The findings from this scan highlight the importance of offering services that provide culturally inclusive care for LGBTQ2S+ youth, and these results can also be used by policy makers to inform policies and procedures in various health care settings including hospitals, community health settings and residential settings. Although the information gathered was specifically on services for LGBTQ2S+ youth, culturally sensitive services must consider the intersecting identities of individuals to truly understand barriers to accessing care. This information will be helpful for informing future research, policies and practice around mental health and substance use care. 

## Figures and Tables

**Table 1:** Number of programs/services per LHIN region, 2017.

LHIN Region	Number of Programs/Services	Programs/Services per 100,000 People *	Programs/Services per 100 Square KM *
Toronto Central	45	3.65	23.4
Hamilton Niagara Haldimand Brant	26	1.86	0.40
Champlain	25	1.93	0.14
Central East	18	1.16	0.12
Mississauga Halton	18	1.55	1.71
Central	17	0.94	0.62
Central West	17	1.84	0.66
South West	16	1.68	0.08
Waterloo Wellington	15	1.96	0.32
Erie St. Clair	12	1.91	0.16
North West	12	5.26	0.01
North Simcoe Muskoka	11	2.37	0.13
North East	11	1.99	0.01
South East	10	2.07	0.05

* Statistics Canada, 2016 Census of Population.

## Data Availability

Data are available on www.smashcovid.ca, accessed on 28 March 2022.

## Abbreviations

LGBTQ2S+	Lesbian, gay, bisexual, trans, queer, 2-spirit, and others
LHIN	Local Health Integration Networks
